# Serum lipopolysaccharide associated with new-onset atrial fibrillation in patients with non-small-cell lung cancer a retrospective observational study

**DOI:** 10.3389/fsurg.2024.1404450

**Published:** 2024-05-09

**Authors:** Haifeng Xu, Jie Zhou, Fei Ye, Yongzhuang Gao

**Affiliations:** Department of Thoracic Surgery, Xuzhou Medical University Affiliated Hospital Sihong Branch, The First People's Hospital of Sihong County, Suqian, Jiangsu, China

**Keywords:** lipopolysaccharide, atrial fibrillation, non-small-cell lung cancer, surgery, new-onset AF

## Abstract

Lipopolysaccharide (LPS) is related to atrial fibrillation (AF). But so far, the relationship between LPS and new-onset AF (NOAF) in patients with lung cancer is unrevealed. This study was to investigate the association between LPS and NOAF in patients after lung cancer surgery. This was a single-center retrospective clinical observational study. Patients diagnosed with non-small-cell lung cancer (NSCLC) were enrolled. All patients receiving lung cancer surgery and at least 24 h electrocardiogram (ECG) examination was recorded during the hospitalization. The incidence of NOAF in this study was 34/406 (8.4%). The univariate analysis showed that NOAF was associated with age, intraoperative blood transfusion (IBT), chronic obstructive pulmonary disorder (COPD), and LPS. After adjusting risk factors, it was found that age, IBT and LPS (OR, 1.031; 95% CI: 1.001–1.042; *P* = 0.002) were still risk factors for NOAF. The area under curve (AUC) value was 0.709 for the LPS. When the LPS was added to the conventional model, the Net reclassification index (NRI) and integrated discrimination index (IDI) were improved significantly. Elevated LPS is associated with an increased risk of NOAF in patients after lung cancer surgery. LPS contributed to the discrimination of the NOAF risk model and improved it markedly.

## 1 Introduction

Cardiovascular diseases (CVDs) are one of the leading causes of death in the population worldwide (1). As one of the cardiovascular disease challenges in the 21st century, atrial fibrillation (AF) is the most common tachyarrhythmia in clinical practice, which can increase the risk of heart failure, stroke and death (2). Recent studies have shown that the prevalence of AF in cancer patients is significantly higher than in the general population. In postoperative lung cancer patients, the prevalence is as high as 8%–42% (1, 3). This is related to a variety of reasons such as cardiac injury and body stress response, in which cancer-related systemic inflammation promotes atrial remodeling and is closely associated with the development of AF (4, 5). Notably, patients with postoperative atrial fibrillation after lung cancer have a poorer prognosis and a higher mortality rate (1). In addition to this, previous studies have shown that patients with a history of cancer are less likely to be treated according to AF guideline recommendations (6, 7). Therefore, exploring more risk factors for the development of atrial fibrillation after lung cancer surgery is of clinical value.

Lipopolysaccharide (LPS), as a component of intestinal flora, has been shown to play an important role in several cardiovascular diseases (8). It is well known that inflammation is one of the central mechanisms of AF, and LPS is a potent trigger of systemic inflammation. A previous animal study showed that LPS activated toll-like receptor 4 (TLR4), which promotes the expression of inflammatory factors, leading to the development of arrhythmias (9). In elderly patients, elevated LPS could promote the development of AF by activating atrial nod-like receptor protein (NLRP)-3 inflammasome (10). Recently, Wang M et al. found that elevated LPS was associated with systemic inflammation and fibrosis biomarkers in patients undergoing AF ablation and was an independent risk factor for AF recurrence during one-year follow-up (11). Notably, LPS could also increase the risk of new-onset AF (NOAF) after cardiac surgery (12, 13). However, the relationship between LPS and new-onset atrial fibrillation (NOAF) after lung cancer surgery has not been revealed. The aim of this study was to evaluate the relationship between LPS and NOAF in postoperative patients with non-small cell lung cancer (NSCLC).

## 2 Methods

### 2.1 Study population

**Figure 1:**
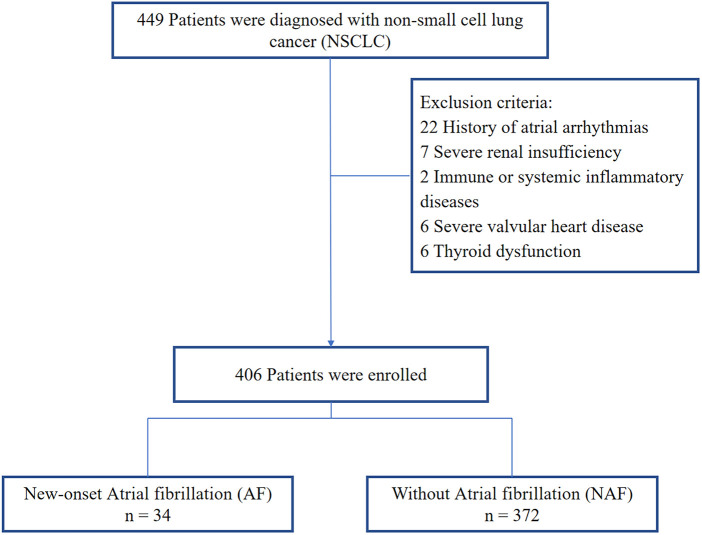
Patient enrollment of the study.

This was a single-center retrospective clinical observational study. We consecutively included patients with NSCLC diagnosed in the Xuzhou Medical University Affiliated Hospital Sihong Branch from January 2020 to December 2023 (14). The Ethics Committee of the Xuzhou Medical University Affiliated Hospital Sihong Branch reviewed this study. All methods were performed in accordance with relevant guidelines and regulations, and written consent was waived owing to the minimal patient risk. Inclusion criteria: lung cancer received surgical treatment; received at least 24 h of cardiac monitoring during hospitalization. Exclusion criteria: history of atrial arrhythmias, severe renal insufficiency, immune or systemic inflammatory diseases, severe valvular heart disease, thyroid dys function. Ultimately, 406 patients who met the eligibility criteria were enrolled (Figure 1).

### 2.2 Data collection and definition of NOAF

We collected all baseline clinical information of enrolled patients, including sex, age, body mass index (BMI), smoking, and past medical history. Fasting venous blood was centrifuged and stored at −80°C until biochemical assays were performed. Serum LPS levels were determined using a commercial ELISA kit (Cusabio, Wuhan, China). LPS standards purified from E. coli and blood samples were incubated for 2 h at room temperature and then dropped onto microtiter plates pre-coated with LPS-specific antibodies. After incubation, samples were read at 450 nm. Intra-assay precision was <8% and inter-assay precision was <10%. Values are expressed in pg/ml. Postoperative heart rates were recorded for all patients. NOAF was defined as at least a 30 s run of AF on electrocardiogram (ECG)/telemetry and no history of AF (15).

### 2.3 Statistical analysis

Statistical analysis was performed using SPSS 26.0 and R software. Kolmogorov–Smirnov method was used for normality test (16). Measures obeying normal distribution were expressed as mean ± standard deviation (SD), and statistical analysis was performed by unpaired *t*-test; measures not normally distributed were expressed as median (Q75, Q75), and statistical analysis was performed by Mann–Whiney *U*-test. Count data were expressed as frequencies or percentages and statistically analyzed using the *χ*^2^ test. Statistically significant variables were included in logistic regression analysis using stepwise forward method was used to analyze the risk factors for NOAF. Receiver operating characteristic (ROC) was used to analyze the predictive value of lipopolysaccharide for NOAF. DeLong test was used to compare the performance of ROC. The net reclassification index (NRI) was calculated based on a comparison between the model's predictions and actual observations (17). NRI and integrated discrimination index (IDI) were used to assess the additional discriminatory power of risk markers. *P* < 0.05 was considered statistically significant.

## 3 Results

### 3.1 Baseline characteristics

The incidence of NOAF in this study was 34/406 (8.4%). Compared with the NAF group, patients with AF were older [(67.55 ± 5.23) years vs. (62.28 ± 6.02) years; *P* = 0.001], with higher C-reactive protein (CRP) [40.5 (12.6, 81.8) mg/L vs. 18.4 (6.6, 56.53) mg/L; *P* = 0.011], heart rate [83 (61.2, 89.0) bpm vs. 72 (62.4, 80.2) bpm; *P* = 0.035] and LPS [(57.37 ± 18.06) ng/ml vs. (42.19 ± 12.27) ng/ml; *P* < 0.001] were significantly higher, with statistically significant differences. Compared with the NAF group, there were significantly higher proportions of chronic obstructive pulmonary disease (COPD) (67.6% vs. 49.5%; *P* = 0.023) and intraoperative blood transfusion (IBT) (17.6% vs. 2.4%; *P* < 0.001) in patients with AF, and the difference was statistically significant (Table 1).

**Table 1:** Patient characteristics.

	AF (*n* = 34)	NAF (*n* = 372)	*P*
Age, years	67.55 ± 5.23	62.28 ± 6.02	0.001
Male, *n* (%)	28 (82.4%)	258 (69.4%)	0.052
BMI, kg/m^2^	22.81 (17.01, 26.22)	22.54 (18.29, 23.38)	0.582
Heart rate, bpm	83 (61.2, 89.0)	72 (62.4, 80.2)	0.035
SBP, mmHg	121 (102,142)	123 (109,141)	0.172
DBP, mmHg	74 (62,83)	75 (67,82)	0.446
CRP, mg/L	40.5 (12.6,81.8)	18.4 (6.6,56.53)	0.011
LPS, pg/ml	57.37 ± 18.06	42.19 ± 12.27	<0.001
Hypertension, *n* (%)	14 (41.2%)	147 (39.5%)	0.721
CAD, *n* (%)	5 (14.7%)	36 (9.7%)	0.402
Heart failure, *n* (%)	2 (5.9%)	15 (4.0%)	0.239
Stroke, *n* (%)	3 (8.8%)	31 (8.3%)	0.923
COPD, *n* (%)	23 (67.6%)	184 (49.5%)	0.023
Diabetes, *n* (%)	8 (23.5%)	80 (21.5%)	0.901
Smoking, *n* (%)	9 (26.5%)	73 (19.6%)	0.282
Left lesion, *n* (%)	11 (32.4%)	148 (39.8%)	0.933
IBT, *n* (%)	6 (17.6%)	9 (2.4%)	<0.001

SBP, systolic blood pressure; DBP, diastolic blood pressure; CRP, C-reactive protein; LPS, lipopolysaccharide; CAD, coronary artery disease; COPD, chronic obstructive pulmonary disease; IBT, intraoperative blood transfusion.

### 3.2 Univariate and multivariate logistic regression analysis

All variables were included in univariate regression analysis, the result showed that NOAF was associated with age, COPD, IBT, and LPS. Inclusion of these variables in multivariate regression analysis using the stepwise forward method revealed that age (OR, 1.102; 95% CI: 1.035–1.180; *P* = 0.001), IBT (OR, 4.211; 95% CI: 1.177–11.518; *P* = 0.037) and LPS (OR, 1.031; 95% CI: 1.001–1.042; *P* = 0.002) were independent risk factors for NOAF during hospitalization in postoperative patients with NSCLC (Table 2).

**Table 2:** Univariate and multivariate logistic regression analysis for new-onset atrial fibrillation.

	Univariate		Multivariate	
	OR (95% CI)	*P*	OR (95% CI)	*P*
Age, years	1.089 (1.032–1.145)	0.001	1.102 (1.035–1.180)	0.001
Male, *n* (%)	2.152 (0.905–4.151)	0.056		
Hypertension, *n* (%)	0.825 (0.451–1.503)	0.592		
CAD, *n* (%)	1.500 (0.423–3.701)	0.328		
Heart failure, *n* (%)	2.120 (0.452–9.89)	0.215		
Stroke, *n* (%)	1.118 (0.322–3.951)	0.833		
COPD, *n* (%)	2.102 (1.114–4.105)	0.032		
Diabetes, *n* (%)	0.830 (0.368–2.072)	0.881		
Smoking, *n* (%)	1.423 (0.751–3.101)	0.211		
Left lesion, *n* (%)	0.993 (0.510–1.934)	0.984		
IBT, *n* (%)	7.071 (2.821–17.354)	<0.001	4.211 (1.177–11.518)	0.037
BMI, kg/m^2^	0.988 (0.891–1.048)	0.602		
Heart rate, bpm	1.028 (1.000–1.042)	0.052		
SBP, mmHg	0.887 (0.876–1.001)	0.060		
DBP, mmHg	0.882 (0.864–1.005)	0.155		
CRP, mg/L	1.023 (0.967–1.001)	0.307		
LPS, pg/ml	1.051 (1.022–1.048)	<0.001	1.031 (1.001–1.042)	0.002

SBP, systolic blood pressure; DBP, diastolic blood pressure; CRP, C-reactive protein; LPS, lipopolysaccharide; CAD, coronary artery disease; COPD, chronic obstructive pulmonary disease; IBT, intraoperative blood transfusion.

### 3.3 Predictive value of lipopolysaccharide for NOAF

**Figure 2:**
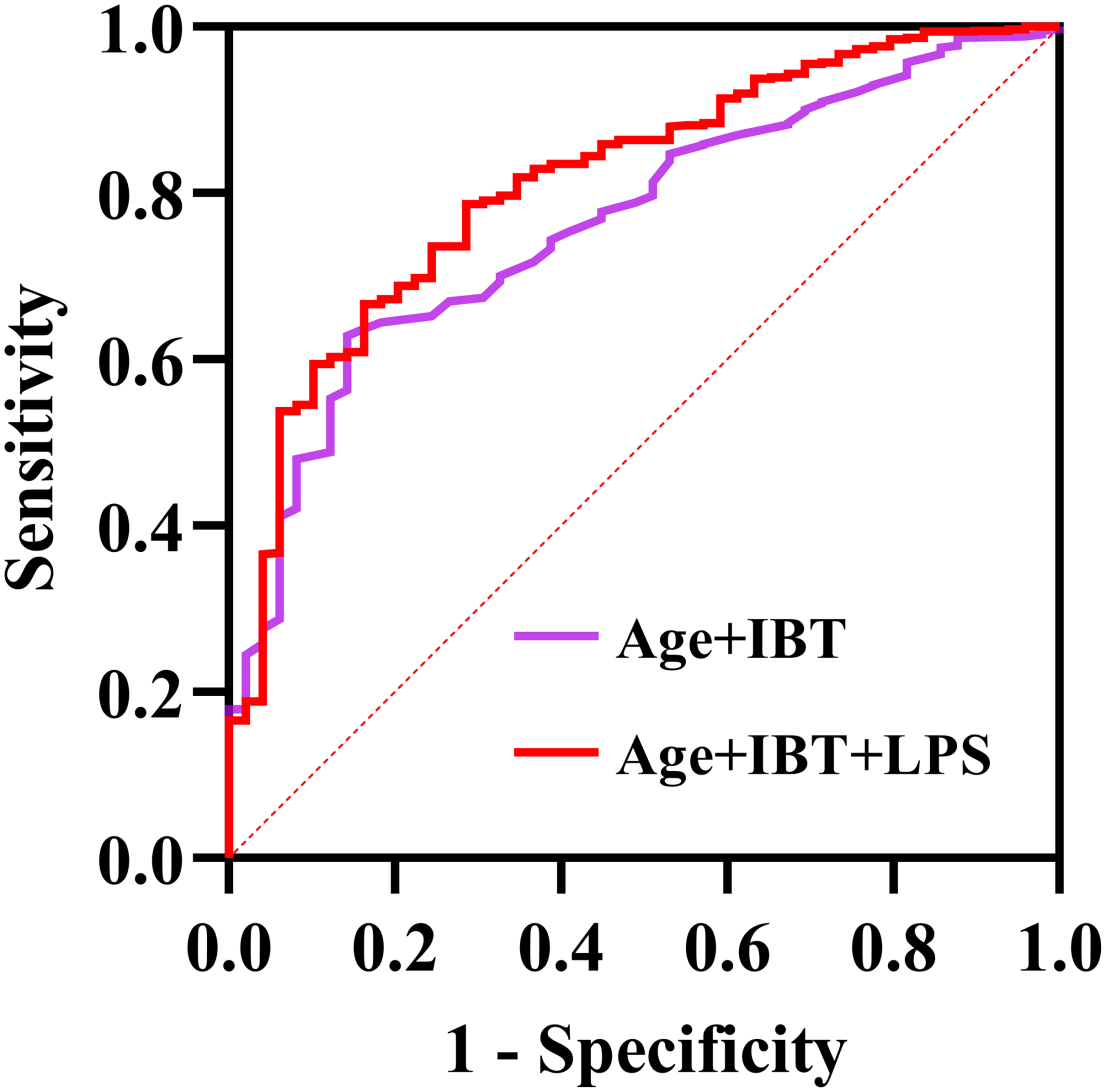
Receiver operating characteristics (ROC) regarding new-onset AF after surgery for non-small cell lung cancer (DeLong test *P*-value was 0.010). AUC, area under the curve; IBT, intraoperative blood transfusion; LPS, lipopolysaccharide.

After incorporating age, IBT and LPS into the ROC, the area under the curve (AUC) values were found to be 0.733 for age, 0.626 for IBT and 0.709 for LPS (Supplementary Figures A–C). The AUC was found to be 0.765 after the joint inclusion of age and IBT in the ROC, and the AUC was 0.814 after the addition of LPS (*P* < 0.001) (Figure 2). DeLong test showed that the diagnostic performance of ROC integrating LPS for NOAF was significantly improved (*P* = 0.010). Based on the results of the multifactorial regression analysis, a traditional model including age and IBT was constructed, and significant improvements in IDI and NRI were observed when LPS was added to the traditional model (Table 3).

**Table 3:** The predictive value of lipopolysaccharide for NOAF.

	AUC (95% CI)	*P*	NRI (95% CI)	*P*	IDI (95% CI)	*P*
Traditional model^a^	0.765 (0.705–0.824)	<0.001	Reference	–	Reference	–
Traditional model + LPS	0.814 (0.756–0.872)	<0.001	0.803 (0.504–1.101)	<0.001	0.035 (0.010–0.061)	0.007

LPS, lipopolysaccharide; NRI, net reclassification index; IDI, integrated discrimination index; AUC, area under the curve.

^a^ Traditional model including age and intraoperative blood transfusion.

## 4 Discussion

To our knowledge, this was the first study on the association between NOAF and LPS in postoperative lung cancer patients. The main findings of this study were: higher LPS was associated with the development of NOAF, and it had a better predictive value for NOAF in postoperative patients with NSCLC. In addition, integration of LPS could significantly improve on the NOAF risk model.

Atrial fibrillation (AF) is one of the most common arrhythmias after pulmonary surgery and is associated with increased mortality, hospitalization time and costs (18, 19). Its pathogenesis is complex and may be related to multiple factors. A notable feature of lung stripping is that after removal of one side of the lung, all pulmonary circulation is forced to flow through the remaining pulmonary vessels, increasing the pressure in the pulmonary veins, which may promote the development of AF. In addition, activation of inflammation can play a further role (20). Previous studies have shown that postoperative NOAF in lung cancer was strongly associated with age (21), heart rate (22), hs-CRP (23), IBT (24) and COPD (25). Consistent with these studies, we found that patients with NOAF were older, had higher hs-CRP, heart rate, and LPS, and greater proportions of COPD and IBT compared with the NAF group. In addition, age and IBT were still found to be associated with NOAF after adjustment for the above risk factors. A previous study found that IBT was a predictor of postoperative NOAF in lung cancer, which is consistent with our findings (3). A possible explanation for this is that an inflammatory immune response is generated by iron and particles in damaged erythrocytes during transfusion (20). Age had been widely recognized as a risk factor for the development of NOAF in patients after surgical procedures (21). Amar et al. (26) evaluated 527 patients who underwent thoracic surgery and they found that patients aged 60 years and older had a significantly higher risk of developing AF. In addition, we innovatively found that LPS was significantly associated with postoperative NOAF in NSCLC patients.

LPS is a cell wall component of Gram-negative bacteria that can cause several cardiovascular diseases once it crosses the intestinal mucosa and enters the body circulation (27). In a prospective study, Pastori et al. (28) found that LPS was significantly associated with major adverse cardiovascular events in patients with AF through increased platelet activation. In addition to this, the association between LPS and AF has been confirmed by studies in patients undergoing radiofrequency ablation of AF as well as in patients undergoing cardiac surgery (11–13). Consistent with these results, we found that LPS and NOAF remained significantly correlated after adjusting for possible confounders. This may be related to the following reasons. It is well known that inflammatory response and oxidative stress were strongly associated with AF, which had likewise been confirmed by many studies in postoperative lung cancer patients (29, 30). It was found that LPS was significantly associated with hs-CRP and IL-6 (11). In an animal study, LPS-TLR4 interaction was revealed to be an important mechanism leading to systemic inflammation and increasing the risk of AF (31). In addition, Menichelli et al. (32) demonstrated in the ATHERO-AF study that LPS can lead to an impaired antioxidant status of the organism. An experimental study have shown that LPS-treated bovine epithelial cells displayed a significant reduction in antioxidant enzyme activities (33). Indeed, LPS has been shown to be involved in multiple pathways leading to increased production of reactive oxygen species in different cardiac diseases (34). These studies suggest that inflammatory responses and oxidative stress may be important mechanisms by which LPS triggers AF. Electrical remodeling and structural remodeling are central mechanisms for the development and maintenance of AF. An animal experiments showed that LPS increased the expression of inflammatory cytokines and l-type calcium channel proteins and shortened the effective atrial occlusion period, thereby promoting the development of AF (30). It has also been reported that LPS, as a potential trigger, could directly alter the function of ionic currents in cardiomyocytes, which together form an arrhythmia axis (35, 36). TGF-β1 is an important marker of atrial fibrosis. Wang M et al. (11) demonstrated a positive correlation between LPS and TGF-β1, and they also found a correlation between the level of LPS and the diameter of the atria. These results may further support that LPS induced NOAF by promoting electrical remodeling and structural remodeling. The present study also found that LPS has a satisfactory predictive value for NOAF, with significant improvements in both IDI and NRI after the addition of LPS to a conventional model about NOAF. In the clinic, the fact that LPS measurements are accurate, reproducible, and available at a reasonable cost seems to provide additional information for NOAF risk assessment in postoperative lung cancer patients.

This study has some limitations. Firstly, it was a single-center retrospective study, which may have some unavoidable bias. Second, this study was conducted on non-small cell lung cancer patients, so the findings may not be applicable to all patients undergoing lung surgery. Third, only patients who received at least 24 h of ECG monitoring were included in this study, which may have introduced some bias. Fourth, although we found that LPS and NOAF were associated, the exact mechanism remains unclear, which may require more basic research to elucidate.

## 5 Conclusion

Elevated LPS is associated with new-onset atrial fibrillation. The addition of LPS can increase the predictability of NOAF in postoperative patients with non-small cell lung cancer.

## Data Availability

The raw data supporting the conclusions of this article will be made available by the authors, without undue reservation.

## Supplementary material

SUPPLEMENTARY FIGURE

Receiver operating characteristics (ROC) regarding new-onset AF after surgery for non-small cell lung cancer. (A) Predictive value of intraoperative blood transfusion for new-onset AF; (B) Predictive value of age for new-onset AF; (C) Predictive value of lipopolysaccharide for new-onset AF. AUC, area under the curve.
