# Supplementary figures and images for: Serum lipopolysaccharide associated with new-onset atrial fibrillation in patients with non-small-cell lung cancer a retrospective observational study
Source: Front Surg. 2024 May 9;11:1404450. doi: 10.3389/fsurg.2024.1404450 (PMC11112106; doi:10.3389/fsurg.2024.1404450)

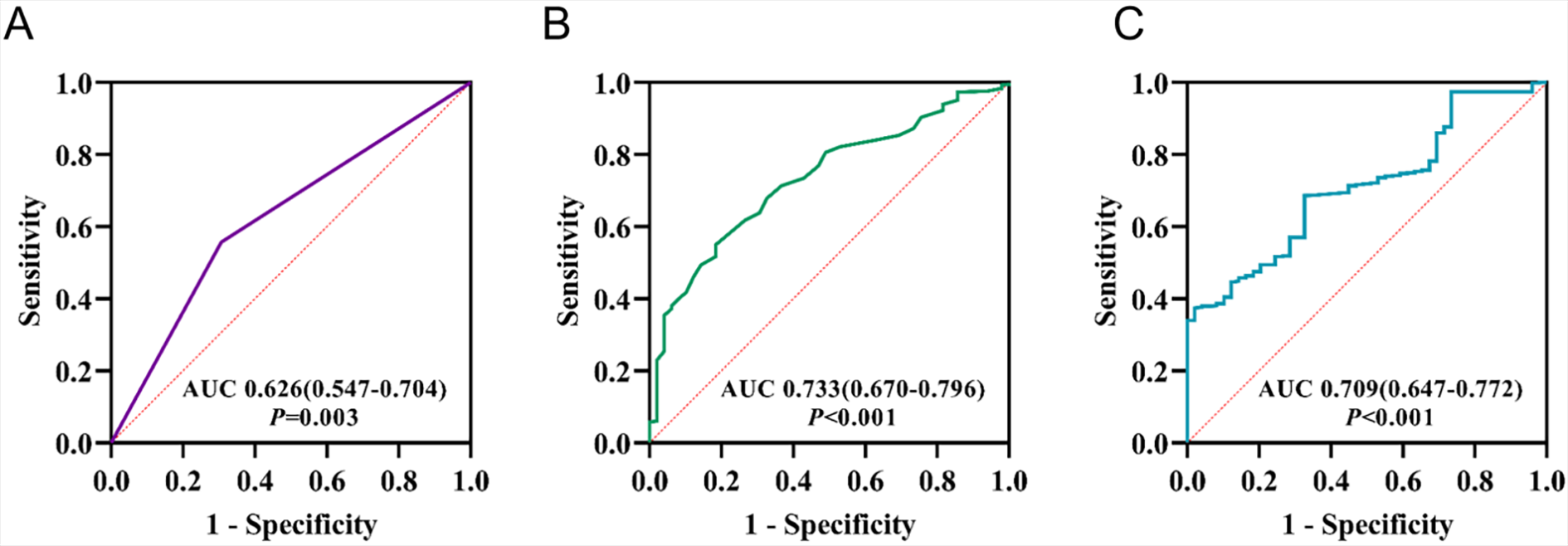

Supplement: Supplementary file 1 [file Image1.tif]
